# Roles of cytoskeleton in metastasis: from its mechanism to therapeutic strategies

**DOI:** 10.1038/s12276-025-01608-9

**Published:** 2026-01-07

**Authors:** Seyeon Lim, Soyeon Woo, Ki Won Lee, Kwang Dong Kim

**Affiliations:** 1 Division of Applied Life Sciences (BK21 Four), Gyeongsang National University, Jinju, Republic of Korea; 2 Department of Molecular and Integrative Physiology, University of Michigan, Ann Arbor, MI USA; 3 Anti-aging Bio Cell Factory-RLRC, Jinju, Republic of Korea; 4 PMBBRC, Gyeongsang National University, Jinju, Republic of Korea; 5 Division of Life Science, Gyeongsang National University, Jinju, Republic of Korea

**Keywords:** Metastasis, Cytoskeleton

## Abstract

The cytoskeleton is a dynamic intracellular protein network composed of actin filaments, microtubules and intermediate filaments that provides structural support in cells and plays a crucial role in tumor metastasis. Tumor cells encounter various dynamic mechanical environments during metastasis, and they adapt to these environments through cytoskeletal reorganization, which enables them to regulate cell morphology, generate intracellular forces and induce intracellular signaling. Actin filaments contribute to migration and extracellular matrix degradation by forming protrusive structures, such as lamellipodia, filopodia and invadopodia. Microtubules support migration, stabilize cell polarity and enhance survival under shear stress. Intermediate filaments maintain structural integrity and mechanical flexibility, allowing cancer cells to pass through narrow spaces. The cytoskeleton’s pivotal role in regulating metastasis makes it a promising drug target. However, cytoskeleton-targeting drugs often face the challenges of nonspecificity and drug resistance. Recent advancements in the field have tried to overcome these limitations through selective targeting, drug delivery systems, antibody–drug conjugates and combination therapies. Here we summarize the roles and regulatory mechanisms of the cytoskeleton in metastasis and discusse the current cytoskeleton-targeting therapies, including their mechanisms, clinical applications and limitations. Furthermore, this review suggests future directions for developing effective and safe cytoskeleton-based interventions against metastasis.

## Cytoskeleton dynamics drive tumor metastasis and therapy advances

Cancer spread, or metastasis, is a major challenge in treating cancer and often leads to death. This process involves cancer cells changing shape and moving through the body. The cytoskeleton, microtubules and intermediate filaments, helps cancer cells move and survive. Researchers have found that targeting the cytoskeleton could help stop cancer spread. However, drugs that target the cytoskeleton can also harm normal cells, causing side effects. Researchers reviewed how the cytoskeleton helps cancer cells spread and discussed current drugs targeting it. They highlighted issues with these drugs, such as toxicity and resistance. The study also explored new strategies to improve these treatments, combining them with other drugs or using advanced delivery systems such as nanoparticles. Researchers concluded that although targeting the cytoskeleton shows promise, more work is needed to make these treatments safer and more effective.

This summary was initially drafted using artificial intelligence, then revised and fact-checked by the author.

## Introduction

Tumor cell metastasis is one of the biggest challenges in cancer treatment and is the main cause of death in most cancer-related mortality in most patients. It is a complex multistep process involving epithelial–mesenchymal transition (EMT), local invasion, intravasation, survival in the circulatory system and colonization of the secondary site. At each step, cancer cells must exhibit remarkable morphological flexibility and functional adaptability to cope with varying mechanical environments. The cytoskeleton is known to play a pivotal role in determining the morphological flexibility of cancer cells^1,2^.

The cytoskeleton is composed of actin filaments, microtubules and intermediate filaments. Each component performs a distinct function, but the components also interact with each other to produce an integrated effect. Beyond simply acting as a structural support, the cytoskeleton plays a key role in regulating cancer cell motility and invasiveness by sensing external mechanical stimuli and transducing them into intracellular signals that lead to morphological changes^3^. During metastasis, tumor cells are exposed to various mechanical stresses, including the stiffness and density of the extracellular matrix (ECM), the physical resistance of surrounding cells during tissue invasion and the shear stress generated during blood circulation. Tumor cells must adapt to these stresses for survival, which they do through the dynamic remodeling of the cytoskeleton. This mechanism allows tumor cells to adjust their shape, stiffness and intrinsic tension according to each metastatic stage. This mechanical adaptability is essential for successful metastasis^4,5^.

As the cytoskeleton has been established as a key regulator of metastasis^6^, it has emerged as a promising therapeutic target for metastasis suppression. However, the development of cytoskeleton-targeting drugs has faced several roadblocks, such as nonspecific toxicity to normal cells, drug resistance and the high adaptive capacity of cancer cells. In this Review, we summarize the various functions performed by the cytoskeleton during metastasis and the underlying molecular mechanisms. We discuss the clinical application and limitations of existing cytoskeleton-targeting drugs. Finally, we also suggest new therapeutic strategies to improve the efficacy and specificity of cytoskeleton-targeting therapy.

## Functions and roles of the cytoskeleton

### Actin filaments

**Fig. 1:**
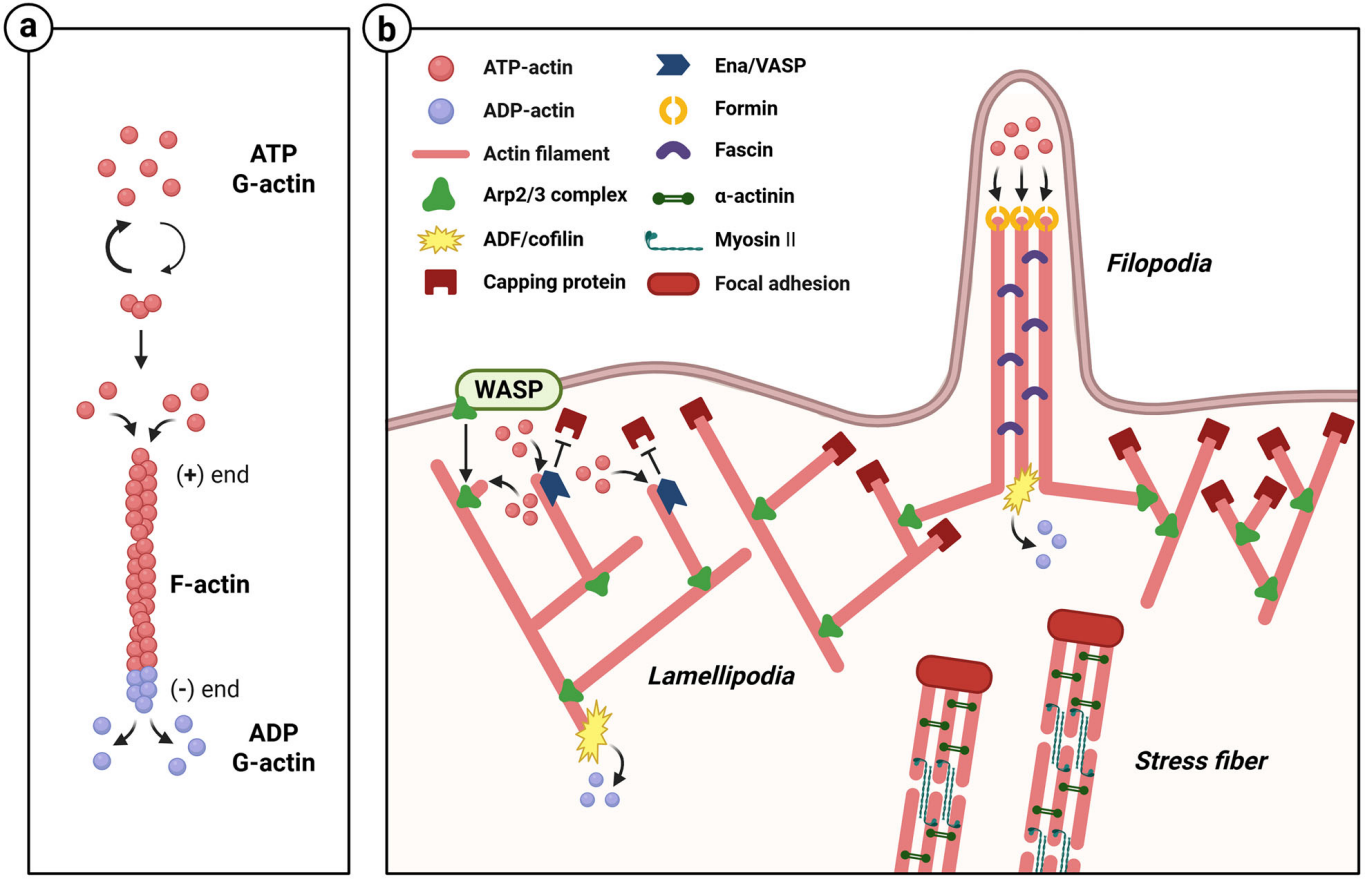
Formation of F-actin and branched and bundled network structures. **a** F-actin is formed through actin nucleation and elongation, initiated by the stable assembly of three ATP-bound actin monomers. **b** F-actin networks are classified into branched and bundled structures. Branched actin networks are nucleated by the Arp2/3 complex, activated by the WASP family. These networks form lamellipodia, enabling broad membrane protrusions. By contrast, bundled actin networks, formed by crosslinking proteins such as fascin and α-actinin, form filopodia and stress fibers.

Actin filaments are the most dynamic cytoskeletal proteins involved in facilitating cell shape change, cell movement and intracellular transport. Actin filaments include globular actin (G-actin) monomers and filamentous actin (F-actin), which is formed through the polymerization of ATP-bound G-actin monomers. The first step in this polymerization process is actin nucleation. This is the rate-limiting step of the process, and filament polymerization proceeds only when G-actin monomers assemble themselves into stable trimers. F-actin has a polarity, with the plus end being bound to ATP-bound G-actin, enabling a rapid and dynamic polymerization. By contrast, the minus end contains a lot of ADP-bound G-actin, causing disassembly and slow growth (Fig. 1a). This polarity of F-actin is vital in determining the direction of cell movement^7,8^.

The Arp2/3 complex and formin (mDia1, mDia2) are actin nucleators that promote actin polymerization. The Arp2/3 complex is activated through interaction with the Wiskott–Aldrich syndrome protein (WASP) family, resulting in the generation of branched networks from unbranched actin filaments. Actin-depolymerizing factor (ADF)/cofilin is a severing protein that preferentially binds to ADP-actin at the minus end of actin filaments, accelerating filament disassembly and promoting actin turnover. The capping proteins, cap Z and gelsolin, bind to the plus end to block actin polymerization. Enabled/vasodilator-stimulated phosphoprotein (Ena/VASP) inhibits capping at the ends of actin filaments via capping proteins, allowing the polymerization to continue. The crosslinkers, fascin and α-actinin, bind to actin, contributing to the formation of actin bundle networks. Parallel actin bundles are composed of actin filaments arranged in the same direction. These are commonly found in filopodia. By contrast, antiparallel bundles, also known as contractile bundles, contain actin filaments oriented in opposite directions. These bundles interact with myosin II to generate contractile forces, contributing to the formation of specific stress fibers^9,10^ (Fig. 1b).

Actin filaments are organized into various structures within the cell. Lamellipodia and filopodia are actin-based protrusion structures that play a crucial role in cell movement. By contrast, blebs are another type of protrusion that regulates cell movement independently of lamellipodia and filopodia. Lamellipodia are a branched actin network composed of Arp2/3 complexes and are broad membrane protrusions located at the leading edge of moving cells that push the cell in the direction of movement^11^. Filopodia are thin, long membrane protrusions that appear at the leading edge of moving cells, transmit intercellular signals and navigate and sense the external environment of the cell^12^. Filopodia extend via formin, with their plus ends pointing toward the cell membrane^13^. Stress fibers form across the cytoplasm as antiparallel bundles linked to α-actinin and myosin II. They are connected to focal adhesions that transmit mechanical forces from the ECM^14^. The actin cortex is a contractile, cross-linked actin network beneath the plasma membrane boundary. It enables cells to sense and respond to external mechanical forces, regulate cortical tension and control cell shape^15^.

### Microtubules

**Fig. 2:**
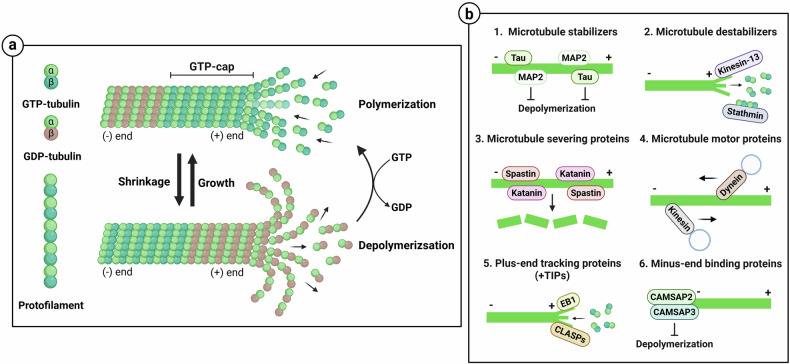
Microtubule dynamic instability and regulation mediated by MBPs. **a** Microtubules are composed of α- and β-tubulin heterodimers. GTP-bound heterodimers polymerize to form protofilaments, which then assemble into microtubules. Microtubule dynamic instability refers to the continuous transition between phases of growth and shrinkage. **b** Various MBPs regulate microtubule polymerization and depolymerization. In the illustration, microtubules are represented by green lines and cargos by blue circles.

Microtubules are composed of α- and β-tubulins and play crucial roles in various cellular processes, such as cell shape maintenance, intracellular transport and mitosis. γ-tubulin of the γ-tubulin ring complex in the microtubule-organizing center acts as a microtubule nucleation factor, interacting with α- and β-tubulin heterodimers to anchor and stabilize them. Then, GTP-bound heterodimers bind and polymerize to form protofilaments, which eventually assemble into a hollow cylindrical structure. Microtubules also exhibit polarity, with the plus end being the polymerization site. Microtubules undergo dynamic instability, characterized by repeated transitions between growth and shrinkage. These transitions are regulated by the GTP cap, which is formed by the addition of GTP-bound β-tubulin to the plus end of microtubules (Fig. 2a). This process stabilizes the microtubules and prevents catastrophe, also known as catastrophic disassembly^16,17^.

Microtubule-binding proteins (MBPs) are classified on the basis of their functions into microtubule stabilizers, destabilizers, severing proteins, motor proteins, plus-end tracking proteins (+TIPs) and minus-end binding proteins. The microtubule stabilizers, tau and microtubule-associated protein 2 (MAP2) bind along the length of the entire microtubule, preventing catastrophic disassembly and stimulating microtubule growth. Conversely, microtubule destabilizers increase microtubule turnover and promote depolymerization. Stathmin binds to tubulin heterodimers to inhibit polymerization, and kinesin-13 promotes microtubule depolymerization by removing the GTP cap in an ATP-dependent manner. The severing proteins, spastin and katanin, promote reorganization by cleaving microtubules into small fragments. Kinesin and dynein are motor proteins that promote intracellular transport along microtubules. Kinesin generally transports cargo to the plus end of microtubules, whereas dynein transports cargo to the minus end. +TIPs such as end binding 1 (EB1) and cytoplasmic linker-associated proteins (CLASPs) bind to the plus end of microtubules and regulate polymerization and microtubule stability^18,19^. By contrast, calmodulin-regulated spectrin-associated proteins (CAMSAPs) bind to the minus end of microtubules. CAMSAP2 and CAMSAP3 stabilize the minus end of microtubules and inhibit depolymerization (Fig. 2b). In addition, posttranslational modifications of microtubules, such as acetylation and detyrosination, can influence microtubule stability by altering the binding affinity and activity of MBPs, thereby regulating microtubule dynamics^20^.

### Intermediate filaments

**Fig. 3:**
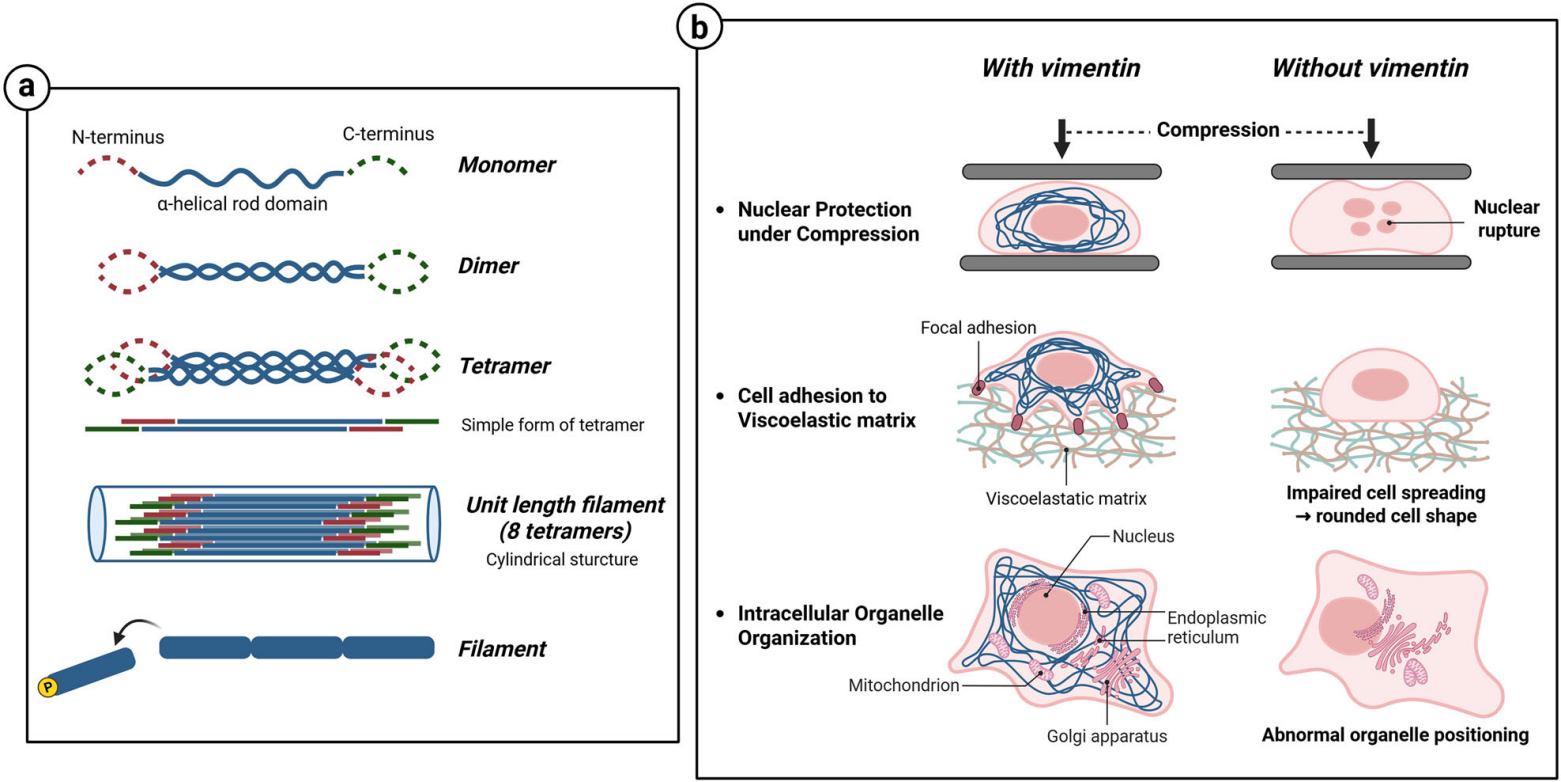
Intermediate filament assembly and integrated roles of vimentin in mechanical stability, cytoskeletal organization. **a** Intermediate filaments are assembled through a stepwise self-assembly process, and the phosphorylation of their subunits induces filament disassembly. **b** Vimentin forms a perinuclear network that protects the nucleus from compressive stress and enhances the cell adhesion to viscoelastic matrices, thereby promoting cell spreading. Vimentin also anchors intracellular organelles and maintains the spatial architecture. In the illustration, vimentin intermediate filaments are depicted as blue lines.

Intermediate filaments are rope-shaped filaments that provide structural integrity and mechanical stability to cells. They assemble through sequential self-assembly without requiring cofactors, ATP or GTP. The monomer structure of intermediate filaments features a central α-helical rod domain, with N-terminal and C-terminal domains on either side. Two monomers combine to form a coiled-coil dimer, and two dimers are arranged antiparallel to form a nonpolar tetramer. A total of eight tetramers combine to create a unit-length filament, a cylindrical structure, and the unit-length filament condenses to form a mature intermediate filament^21^. Unlike other cytoskeletal components that depend on nucleotide hydrolysis for polymerization and depolymerization, the dynamics of intermediate filaments are primarily regulated through phosphorylation-dependent reorganization. The phosphorylation of intermediate filaments disrupts the lateral interactions between intermediate filament subunits, promoting the disassembly and reorganization of filaments. This dynamic process is essential for cellular functions, such as migration, mitosis and smooth muscle contraction^22^ (Fig. 3a).

Intermediate filaments are classified into five types. Types I and II are acidic and basic keratins combined to form heterodimer filaments. They provide structural integrity to epithelial cells and protect cells from mechanical stress. Type III comprises vimentin, desmin, glial fibrillary acidic protein and peripherin, which combine to form homodimeric or heterodimeric filaments. Type IV, a neurofilament (NF), which includes NF-light (NF-L), NF-medium (NF-M), NF-heavy (NF-H) and α-internexin, is important for axon stability. Type V is a nuclear intermediate filament composed of lamins A, B and C, which collectively maintain the shape of the nucleus and prevent damage to it^17^.

Intermediate filaments play a pivotal role in mitigating cellular damage caused by external mechanical forces^23^. Vimentin forms a dense perinuclear network that protects the nucleus from mechanical compression. Unlike other cytoskeletal components, vimentin networks critically contribute to compression stiffening by enabling cells to resist the mechanical constraints encountered during migration through confined spaces^24^. Furthermore, vimentin enhances cellular adhesion with viscoelastic matrices, promoting cell spreading and an adaptive morphological response. These adhesion effects may influence stress fiber organization; however, the precise relationship between vimentin and stress fiber assembly remains unclear^25^. In addition to its mechanical functions, vimentin contributes to the spatial organization and maintenance of intracellular architecture by anchoring organelles such as the Golgi apparatus, the endoplasmic reticulum, the nucleus and the mitochondria^26^ (Fig. 3b). Moreover, the interplay between vimentin intermediate filaments and actin filaments is crucial for cell adhesion, motility and invasiveness. Plectin is a protein that connects actin and intermediate filaments, and the inhibition of plectin disrupts the connection between vimentin and F-actin in invadopodia, which are thin actin-rich protrusions necessary for matrix degradation, thereby inhibiting invadopodia formation and subsequent metastasis^27,28^. Vimentin regulates actin cytoskeletal organization and cell motility through its association with capping protein regulator and myosin 1 linker 2 (CARMIL2). CARMIL2 regulates actin filament elongation by inhibiting capping protein and indirectly affects the activity of Arp2/3 complex, promoting invadopodia formation and cell motility^29^.

## Role and mechanism of the cytoskeleton in the tumor cell metastasis

Cancer cells encounter new physical environments during metastasis. High-stiffness ECM, narrow gaps between vascular endothelial cells, shear stress due to blood flow and intercellular adhesion resistance act as physical obstacles to the movement and survival of cancer cells. To actively respond to such external stresses, cancer cells must sense and adapt to external stimuli. The sophisticated remodeling of the cytoskeleton forms the cornerstone of this adaptive response.

### In the EMT and invasion of tumor cells

**Fig. 4:**
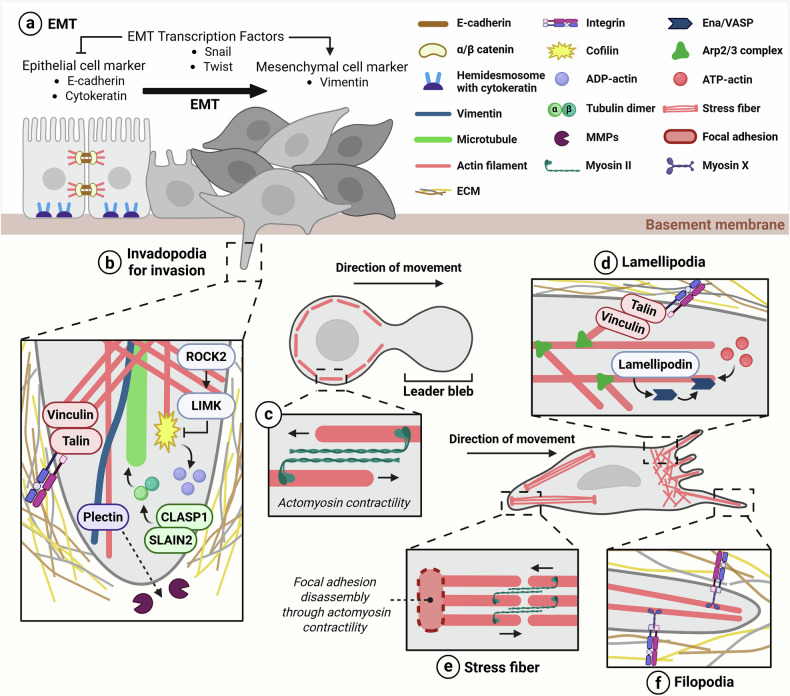
Cancer cell migration and invasion through specialized protrusions and cytoskeletal reorganization. **a** Epithelial cells have strong cell-to-cell adhesion through adherens junctions and hemidesmosomes. The EMT transcription factors suppress the expression of epithelial cell markers such as E-cadherin and cytokeratin and induce the expression of mesenchymal cell marker such as vimentin during the EMT process. **b** The invadopodia promote cell invasion by increasing ECM degradation via MMPs, which are anchored to the plasma membrane by binding to integrins via talin and vinculin. The ROCK2–LIMK pathway regulates the stability of invadopodia by inhibiting cofilin. CLASP1 and SLAIN2 promote the microtubule growth. Plectin reinforces invadopodia stability by linking intermediate filaments to actin filaments. **c** The leader bleb is formed owing to increased pressure from actomyosin contraction at the back of the cell, driving the cell migration. **d** The lamellipodia enhance the cell motility through actin polymerization, mediated by Ena/VASP interacting with lamellipodin. **e** The myosin II contraction of stress fibers at the posterior of mesenchymal cells induces focal adhesion disassembly and cell migration. **f** The filopodia recognize the extracellular environment by binding to integrins via myosin-X. ROCK2, rho-associated coiled-coil protein kinase 2.

EMT is an early stage of metastasis, a process through which cancer cells transition from an epithelial morphology with strong cell-to-cell adhesion to a more detached and invasive mesenchymal morphology. E-cadherin, expressed in epithelial cells, binds directly to β-catenin and α-catenin, as well as indirectly to actin filaments. When EMT is induced, Snail and Twist repress the expression of E-cadherin, and its binding to α/β-catenin is weakened, leading to the disintegration of the circumferential actin bundle that maintains cell-to-cell adhesion^30,31^. Epithelial cells mainly express cytokeratin intermediate filaments, which interact with hemidesmosomes to strengthen adhesion to the basement membrane, contributing to the maintenance of cell morphology and structural stability. When EMT is induced, Snail suppresses cytokeratin expression while increasing vimentin expression^32,33^ (Fig. 4a). The accumulating vimentin forms an extensive network within the cell and interacts with actin filaments and microtubules to increase cell motility and invasiveness.

During invasion, tumor cells encounter a dense ECM that impedes cellular penetration. To overcome this challenge, cancer cells remodel actin filaments to form protrusive structures such as invadopodia. Invadopodia are composed of a central F-actin-rich actin core surrounded by a ring region. The actin core forms the protrusion of the cell, allowing it to pass through the ECM, and the ring region surrounding the actin core contains proteins such as vinculin and talin, which are involved in adhesion to the ECM, thereby increasing the adhesion and structural stability of the invadopodia^34^. Phosphorylated cofilin by LIM kinase (LIMK) loses its actin-severing activity, thus inhibiting the disassembly of actin filaments and stabilizing the structure of invadopodia^35^. Invadopodia also contain ECM-degrading proteins, such as membrane-type matrix metalloproteinase (MMP)2 and MMP9, which facilitate ECM penetration^36^.

SLAIN motif-containing protein 2 (SLAIN2) is a protein that recruits +TIPs to microtubules, forming a complex with CLASP1 to accelerate microtubule polymerization at the growing ends^37^ (Fig. 4b). The ends of the protrusions bind to collagen through integrins, enabling cell movement^38^. The increased deacetylation of α-tubulin in prostate cancer increases the stability of microtubules by making them resistant to depolymerization. Moreover, phosphorylation of stathmin in cancer cells decreases its binding to tubulin, which attenuates its depolymerization activity and hence increases microtubule stability^39,40^. Vimentin affects cancer cell invasion by stabilizing the actin core of invadopodia through binding with plectin and F-actin, which results in the maintenance of invadopodia polarity and lamellipodia formation^28,41^. Vimentin also enhances mechanical stability, allowing cells to deform and migrate through confined spaces while withstanding intracellular pressure, which is advantageous during invasive migration^42,43^.

### In the migration of tumor cells

Depending on the ability of the cytoskeleton to modulate cellular flexibility and overcome mechanical constraints, tumor cells selectively adopt two primary types of movement: amoeboid and mesenchymal. Amoeboid movement is characterized by rounded cell shapes, low adhesion to the ECM and rapid migration through confined spaces. This mode relies on high actomyosin contractility, in which myosin II and actin filaments generate the intracellular pressure that drives membrane protrusions. A hallmark of amoeboid migration is the leader bleb: a pressure-driven protrusion at the front of the cell. In this mode, Arp2/3 complex activity is suppressed, whereas the formation of linear actin bundles is increased to support cortical tension and blebbing dynamics. Importantly, amoeboid migration involves minimal ECM degradation and enables rapid, shape-adaptive migration through narrow tissue spaces^44–46^ (Fig. 4c).

By contrast, mesenchymal movement features elongated cell morphology, strong integrin-mediated adhesion to the ECM and protease-dependent ECM remodeling. This migration type is driven by actin-based protrusions, such as lamellipodia and filopodia, which form at the leading edge. Lamellipodia with branched actin networks are driven by high Arp2/3 complex activity^44^. Lamellipodia attach to the ECM through focal adhesions involving talin and vinculin, generating pulling forces for forward movement^47^. Actin filaments in lamellipodia interact with lamellipodin to promote cancer cell migration. Lamellipodin recruits Ena/VASP, which then allows it to attach to actin filaments, promoting actin polymerization^48^ (Fig. 4d). Overexpression of the M and A isoforms of microphthalmia-associated transcription factor (MITF) in the melanoma cell line SK-MEL-24 promoted actin polymerization, thereby increasing lamellipodia formation and cell mobility^49^. Simultaneously, myosin II-mediated contraction of stress fibers at the rear edge generates tension that drives forward movement. These contractions promote the focal adhesion disassembly, allowing the cell to move forward better^50^ (Fig. 4e). Filopodia, formed by the reorganization of actin filaments within lamellipodia, are used by cells to recognize their surroundings through myosin^51,52^. Myosin-X, one of the regulators of filopodia movement, contains four-point-one, ezrin, radixin and moesin (FERM) domains that bind to integrins to form filopodia adhesion with the ECM, facilitating cell movement^53^ (Fig. 4f).

Amoeboid and mesenchymal migration modes differ in their cytoskeletal organization, adhesion strength and dependency on ECM degradation. Amoeboid migration is rapid, low adhesion and protease-independent, whereas mesenchymal migration is slower, adhesive and reliant on ECM remodeling. Cancer cells can interconvert between amoeboid–mesenchymal and mesenchymal–amoeboid transitions to rapidly adapt to their environment and maximize their ability to survive and metastasize^44^.

### In the intravasation of tumor cells

**Fig. 5:**
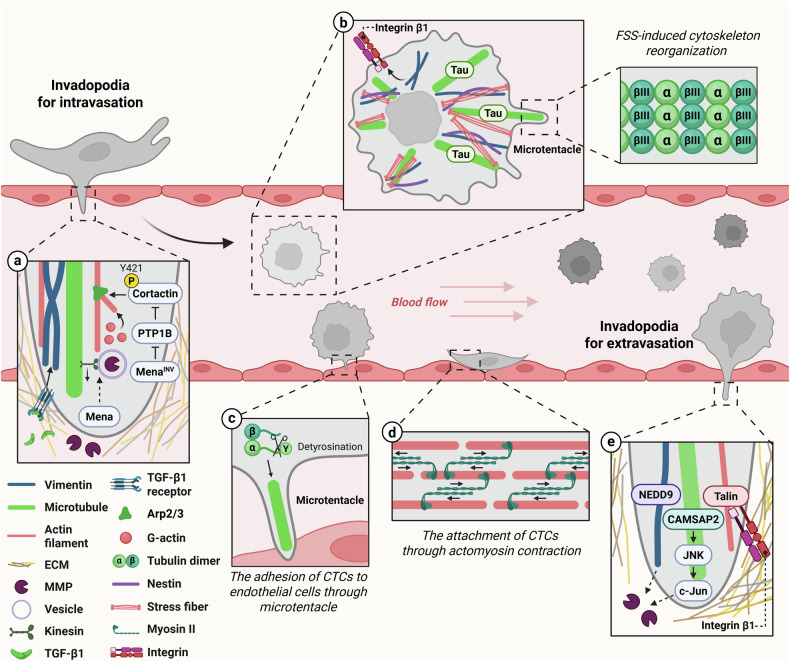
Intravasation and extravasation of cancer cells and mechanical resistance of CTCs via cytoskeletal modification and reorganization. **a** Mena^INV^, an isoform of Mena that increases during intravascular penetration, stabilizes actin filaments through PTP1B inhibition. Mena promotes the secretion of MMPs through interaction with microtubules. The increased expression of TGF-β1 upregulates vimentin to enhance the stability of the cytoskeleton. **b** CTCs express βIII-tubulin and Tau to form and stabilize microtentacles and reorganize the actin cytoskeleton into stress fibers. Vimentin increases the cell surface expression of integrin β1, which allows cell survival. **c** Microtentacles, formed by detyrosination of α-tubulin, facilitate the attachment of CTCs to endothelial cells. **d** The actomyosin contractility enhances the adhesion of CTCs to endothelial cells for extravasation. **e** During extravasation, invadopodia mature through integrin β1 and talin binding, increase MMP expression by CAMSAP2 and promote MMP secretion through the interaction of NEDD9 with vimentin, allowing cancer cells to pass through endothelial cells easily.

During intravasation, the invadopodia play a crucial role in tumor cell invasion by enhancing the protrusion ability of tumor cells through maturation and structural stabilization. These processes are mediated by an alternative splice variant of mammalian-enabled protein (Mena) called mammalian-enabled invasive isoform (Mena^INV^)^54,55^. Mena^INV^ inhibits protein tyrosine phosphatase 1B (PTP1B), stabilizing the phosphorylation of cortacin at Tyr421. This, in turn, promotes the activation of the Arp2/3 complex and the stabilization of invadopodia^56^. Mena enhances the recycling of MMP within the cell, enabling MMP-containing vesicles to travel along microtubules to invadopodia via kinesin^57^ (Fig. 5a). In the tumor microenvironment of metastatic cells, the interplay of microtubules in both cancer cells and endothelial cells contributes to the loosening of endothelial cell junctions, allowing the cancer cells to penetrate blood vessels^58^. During intravasation, the expression of transforming growth factor beta 1 (TGF-β1) increases, which leads to the upregulation of vimentin. Vimentin strengthens cytoskeletal integrity and enhances cell stiffness, providing the mechanical support necessary for endothelial barrier penetration^59^.

### In the circulation of tumor cells

Circulating tumor cells (CTCs) face harsh mechanical forces such as fluid shear stress (FSS) and dynamic pressure within the bloodstream. To overcome these challenges, CTCs reorganize their actin filaments, increasing stiffness through actin polymerization and stress fiber formation, which confers resistance against elevated blood pressure^60^ (Fig. 5b). The resistance to FSS during circulation is achieved through the formation of microtentacles. The overexpression of a specific isoform of β-tubulin, βIII-tubulin, alters microtubule dynamics, allowing CTCs to survive^61^. Tau enhances the formation of microtentacles in detached cancer cells by stabilizing microtubules and promoting cellular reattachment during the circulation^62^. Vimentin, which stabilizes the cytoskeleton, also enables cancer cells to adapt to FSS. Vimentin dissipates the force generated inside the cell during cell transformation, thereby preventing damage to the nucleus and cell membrane, allowing resistance to FSS^63^. Simultaneously, vimentin enhances integrin β1 expression to the cell surface, allowing integrin-mediated clustering of suspended cells^64^. This increased cell adhesion may allow CTCs to better survive in the bloodstream. Nestin regulates cell stiffness through its interaction with vimentin, and when nestin expression is reduced, cells cannot withstand the bloodstream pressure, resulting in the induction of anoikis^65^.

### In extravasation of CTCs

During extravasation, CTCs navigate compressive forces and physical constraints while passing through endothelial cell junctions and narrow capillaries. The detyrosinated form of α-tubulin, which is promoted by histone deacetylases (HDAC), enhances the adhesion of CTCs to endothelial cells in the bloodstream^66^ (Fig. 5c). CTCs bind to platelets to form small protrusions that help them better adhere to endothelial cells and interact with surrounding immune cells, giving them an advantage in escaping the blood vessel^67^. Cancer cells utilize actomyosin contractility to adhere to and migrate to vascular endothelial cells, enabling extravasation^68^ (Fig. 5d). This morphological adaptation is driven by cytoskeletal contractility, a biomechanical response that allows cells to overcome spatial constraints.

Invadopodia are active during extravasation and secrete MMPs, which allow tumor cells to penetrate the endothelial cell barrier and metastasize to other sites. Extravasation begins as the gap in the endothelial cells widens^69^. Talin, which binds both integrin β1 and actin filaments, probably contributes to invadopodium stabilization by anchoring the precursor structure to the ECM, promoting the initiation of the maturation process^70^. CAMSAP2 promotes the migration and invasion of cancer cells by increasing the expression of MMP-1 through the activation of the c-Jun N-terminal kinase (JNK)–c-Jun pathway^71^. During extravasation, vimentin rearranges its structure to better withstand the pressure generated when tumor cells pass between endothelial cells. Conversely, when vimentin expression is reduced, cells lose their resistance to pressure, and their extravasation ability is compromised^28^. Vimentin interacts with neural precursor cell expressed developmentally downregulated protein 9 (NEDD9) in cancer cells, and this interaction promotes MMP9 secretion and increases cancer cell invasiveness^72^ (Fig. 5e). In a lung metastasis model of metastatic melanoma, disruption of the vimentin–NEDD9 complex using a vimentin inhibitor notably reduced lung metastasis^73^. Therefore, the vimentin–NEDD9 axis can be considered to have an important impact on cancer cell invasiveness and metastasis. In addition, vimentin is connected to invadopodia through plectin, and the formation of this structure is essential for ECM degradation and the passage of tumor cells through the endothelial barrier^28^.

## Anticancer agents targeting the cytoskeleton of cancer cells

The cytoskeleton plays an essential role in cell division, movement and intracellular transport, and its abnormal regulation is closely associated with cancer progression and metastasis. Naturally, then, the development of anticancer drugs targeting the cytoskeleton of cancer cells has become one of the hotspots in cancer research.

### Actin filament-targeting agents

Actin filaments have attracted attention as potential targets for antimetastatic drugs because they play a fundamental role in cancer cell motility and metastasis-associated invasiveness. Actin-targeting drugs disrupt actin dynamics by stabilizing or destabilizing actin. Given that actin filaments are essential components of normal cells, actin-targeting drugs often have nonspecific effects and fatal adverse effects, such as cardiac and renal toxicity and liver damage. Thus, the development of these drugs has been limited to the preclinical stage^74^, and no drugs have currently received FDA approval. Recently, active research is being conducted to discover new actin-regulating compounds that selectively act on cancer cells and have minimal effects on normal cells.

Cytochalasin binds to F-actin and inhibits actin polymerization. Although it effectively inhibits lung metastasis in melanoma metastatic models^75^, its clinical utility is limited by its adverse side effect of causing cardiotoxicity, which results in reduced cardiac contractility^76^. Latrunculin binds to G-actin, inhibits actin polymerization and increases depolymerization to promote actin filament disruption. However, the continuous perfusion of latrunculin was found to induce seizures in a rat model^77^. Conversely, jasplakinolide stabilizes actin filaments by promoting actin polymerization. It inhibited the growth of human prostate cancer cells and reduced lung cancer metastasis^78^. However, the side effects of jasplakinolide include compromised cardiomyocyte function and cardiotoxicity^79^. Chondramide, a cyclodepsipeptide produced by *Chondromyces crocatus*, was found to inhibit breast cancer cell migration, invasion and lung metastasis in an in vivo model without inducing toxicity^80,81^. The mechanism involves the promotion of abnormal F-actin aggregation and a decrease in cell contractility due to the inactivation of rho GTPase. Furthermore, chondramide inhibits actin dynamics and induces actin aggregation in breast cancer cells overexpressing protein kinase C epsilon (PKCε). PKCε, which can bind to actin, gets trapped in abnormally aggregated actin bundles, which weakens PKCε activity and induces apoptosis. PKCε is a cell survival regulator and is often upregulated in aggressive cancers. The apoptotic effect of chondramide is absent in nontumorous breast epithelial cells, such as MCF-10A. These cells have low PKCε expression, suggesting that chondramide-induced apoptosis is dependent on PKCε expression. Overall, chondramide inhibits tumor growth and metastasis by impairing actin-mediated cytoskeletal reorganization and promoting cancer-specific apoptosis through PKCε inactivation^82^. TR100 is an antitropomyosin compound that targets the Tm5NM1 and Tm5NM2 isoforms of tropomyosin, which are selectively overexpressed in various cancer cells. Tropomyosin binds to actin filaments and stabilizes them by inhibiting actin depolymerization. TR100 selectively destabilizes actin filaments in malignant tumor cells by interfering with the interaction between Tm5NM1/2 and actin filaments, thereby reducing the motility and survival of cancer cells. In addition, in vivo studies have demonstrated that TR100 effectively inhibits the growth of melanoma and neuroblastoma without causing hepatotoxicity and cardiotoxicity, which are common side effects of existing actin-targeting drugs. These results showcase the potential of TR100 as an actin-targeting drug that can block tumor metastasis with minimal toxicity^83,84^.

As the direct inhibition of actin polymerization has been associated with high toxicity in normal tissues, recent studies have focused on indirect approaches involving the modulation of upstream regulators of actin dynamics. Among these, the inhibition of rho-associated coiled-coil protein kinase (ROCK) and LIMK has shown potential in selectively targeting the metastatic potential of cancer cells. ROCK is a key effector of the rho signaling pathway, regulating actomyosin contractility by phosphorylating myosin light chain kinase (MLCK) and LIMK. The activation of ROCK plays a pivotal role in cell migration, invasion, focal adhesion and stress fiber formation. Fasudil, a clinically approved ROCK inhibitor for cerebrovascular disease in Japan and China, was found to inhibit ovarian cancer cell motility and invasion by suppressing ROCK activation and interfering with cytoskeletal rearrangements, such as the lysophosphatidic acid-induced formation of stress fibers and focal adhesions. Fasudil reduced intraperitoneal tumors in an ovarian cancer xenograft model^85^. Y-27632, the first selective ROCK inhibitor, was found to inhibit the ROCK–MCLK signaling pathway in bladder cancer, thereby reducing actomyosin-mediated contractility and inhibiting the proliferation and invasion of bladder cancer cells^86^. CCT129254, another ROCK inhibitor, was shown to reduce both the migration and invasion of amoeboid and mesenchymal-like melanoma cells by inhibiting ROCK-induced actomyosin contractility. CCT129254 also effectively suppressed melanoma lung metastasis in vivo without affecting primary tumor growth^87^. LIMK, a downstream effector of ROCK, phosphorylates and inactivates cofilin, thereby promoting the stabilization of actin filaments. Increased LIMK activity, frequently observed in cancer cells, promotes the formation of protrusions such as lamellipodia and filopodia, thereby increasing cancer cell migration and invasion. Preclinical studies using LIMK inhibitors, such as 4-pyridocarbazolone (Pyr1), have demonstrated the effective inhibition of tumor cell motility and metastasis. LIMK inhibition by Pyr1 promotes actin depolymerization via cofilin and reduces the formation of invasive protrusion structures. Pyr1 was found to inhibit tumor growth and metastasis in breast cancer xenograft models^88^. As ROCK and LIMK inhibitors disrupt critical components of actin remodeling, targeting these upstream regulators of actin dynamics may be a selective strategy to efficiently inhibit metastasis by targeting the cytoskeleton.

### MTAs

Among cytoskeletal components such as actin filaments, microtubules and intermediate filaments, only drugs that directly target microtubules have received FDA approval. Microtubules possess structural and functional properties that make them advantageous targets for anticancer drugs. Because they are essential for spindle formation, they are highly effective targets for selectively inhibiting rapidly dividing cancer cells^89^. Therefore, microtubules have emerged as important targets for developing cytotoxic anticancer drugs. Microtubule-targeting agents (MTAs) are primarily classified into microtubule-stabilizing and microtubule-destabilizing agents. Representative microtubule-stabilizing agents approved by the FDA include taxane anticancer drugs, such as paclitaxel and docetaxel, which promote microtubule polymerization and stabilization. Paclitaxel binds to β-tubulin and promotes tubulin polymerization. It has been used to treat various cancers, including breast and lung cancer^90–92^. Docetaxel, developed as a second-generation taxane drug, is used to treat breast, lung and prostate cancer^93^. Representative microtubule-destabilizing agents, such as vinca alkaloid anticancer drugs vincristine and vinblastine, are also approved by the FDA, which inhibit microtubule polymerization and cause microtubule destabilization^94,95^. Vincristine and vinblastine destabilize microtubules and induce apoptosis by binding to the vinca site of β-tubulin. Clinical success in the use of vincristine and vinblastine has been achieved in hematological malignancies, including lymphoma and breast cancer^96,97^.

The continued therapeutic use of MTAs may result in increased drug resistance and adverse effects. Cells commonly develop multiple drug resistance by exporting the therapeutic agent out of the cell. P-glycoprotein, an ATP-binding cassette transporter, helps export taxanes out of cancer cells, thus counteracting the protective effects of taxanes over time^98^. Cabazitaxel was developed as a strategy to overcome the drug resistance. Cabazitaxel is a semisynthetic analog of docetaxel with low affinity for P-glycoprotein and can maintain antitumor activity even in cancer cells resistant to existing docetaxel. Cabazitaxel was approved by the FDA in 2010 in combination with the anti-inflammatory drug prednisone for patients with metastatic castration-resistant prostate cancer who had failed docetaxel treatment^99^. Several clinical studies have shown that cabazitaxel is highly effective even in patients with metastatic breast cancer resistant to taxane drugs^100^. Ixabepilone is a semisynthetic analog of epothilone B that inhibits mitosis by stabilizing microtubules. As this drug has a low binding affinity for P-glycoprotein, it remains effective even in cancer cells resistant to existing MTAs. In 2007, Ixabepilone received FDA approval as monotherapy or in combination with capecitabine for patients with metastatic or locally advanced breast cancer who have failed taxane therapy^101^. However, peripheral neuropathy and bone marrow suppression have been reported as major side effects, and dosage adjustment may be necessary depending on the patient’s condition^102^.

### Intermediate filament-targeting agents

Intermediate filaments exhibit tissue-specific expression and constitute a major portion of cytoskeletal proteins in mesenchymal cells. Overexpressed vimentin is found in various cancers, and vimentin expression is inversely correlated with disease prognosis, making it a potential prognostic biomarker^103^. Withaferin A is a vimentin inhibitor that suppresses tumor growth and angiogenesis. Withaferin A inhibited capillary growth in a corneal angiogenesis model, but this inhibitory effect was attenuated in vimentin-deficient mice. Moreover, withaferin A was found to induce proteasome-mediated degradation of NF-κB, inhibit its activity and thereby reduce cyclin D1 expression in a human umbilical vein endothelial cell (HUVEC) model^104^. Combination therapy with withaferin A and sorafenib produced a synergistic effect in suppressing the growth of thyroid cancer, and the potent anticancer effect was observed even at a lower dose of sorafenib^105^. Silibinin was shown to inhibit the migration and invasion of prostate cancer cells by downregulating vimentin and MMP2^106^. Although vimentin offers the potential for developing selective anticancer agents to treat mesenchymal cancers, no intermediate filament-targeting anticancer agents are currently in clinical trials. Because intermediate filaments are involved in normal physiological processes, such as wound healing and immune responses, the inhibition of them may also affect the function of normal tissues^107^. Furthermore, intermediate filaments lack polarity, exhibit structural diversity and have poorly defined binding sites for small molecule compounds. Because of these structural and biological constraints, no anticancer drugs that directly target intermediate filaments, such as actin, have been approved by the FDA so far.

## Novel cytoskeleton-targeting strategies for tumor therapy

### Combination therapy using cytoskeleton-targeting drugs and other anticancer drugs

Microtubule stabilizers can increase immune cell infiltration. Meanwhile, the evasion of the immune system through immune tolerance has been reported in various cancer types. Therefore, several studies have explored the effectiveness of combination therapy using microtubule stabilizers and immunotolerance suppressors, such as antagonists of programmed cell death protein 1 (PD-1)/programmed death ligand 1 (PD-L1), in various cancer types (Table 1). A combination therapy of paclitaxel, which inhibits cancer cell division by stabilizing polymerized microtubules, and pembrolizumab, an antagonist of PD-1, prolonged the progression-free survival period in patients^108^. Moreover, a phase 3 clinical trial was also conducted on a triple combination therapy of paclitaxel, tiragolumab and atezolizumab. Triagolumab inhibits the T cell immunoreceptor with immunoglobulin and immunoreceptor tyrosine-based inhibitory motif (ITIM) domains, whereas atezolizumab enables immune cells to attack cancer cells by neutralizing PD-L1^109^.

**Table 1:** Combination therapy with monoclonal antibodies or DNA-damaging drugs and cytoskeletal targeting drugs.

Drug	Mechanism and features	Clinical progress	Reference
Paclitaxel–pembrolizumab	Paclitaxel: inhibits division of cancer cells by stabilizing polymerized microtubules and inhibiting their degradation to tubulin Pembrolizumab: activates immune response by inhibiting PD-1 receptors on the surface of cancer cells	Phase 2	^108^
Tiragolumab–paclitaxel–atezolizumab	Tiragolumab: binds to TIGIT and inhibits cancer cell evasion of the immune system Atezolizumab: binds to PD-L1 and inhibits the interaction between PD-1 and CD80 receptor	Phase 3	^109^
Paclitaxel–bevacizumab	Bevacizumab: blocks angiogenesis by inhibiting VEGF	FDA approved	^110,111^
Docetaxel–ramucirumab	Docetaxel: stabilizes cellular microtubules by interfering with tubulin depolymerization Ramucirumab: inhibits VEGF receptor 2	NSCLC: FDA approved Gastric cancer: phase 2	^112,113^
CA4P–carboplatin/paclitaxel–carboplatin	CA4P: targets tubulin to interfere with microtubule assembly Carboplatin: inhibits DNA synthesis by inhibiting interstrand crosslink formation in DNA	Phase 2/FDA approved	^115,117,118^
Paclitaxel–durvalumab–olaparib	Durvalumab: blocks PD-L1 in cancer cells as a PD-L1 inhibitor Olaparib: blocks DNA repair by blocking the action of Poly (ADP-ribose) polymerase	Phase 2	^116^

The combination therapy of paclitaxel and bevacizumab suppresses neovasculogenesis by inhibiting vascular endothelial growth factor (VEGF). This combination therapy was found to extend progression-free survival compared with monotherapy, resulting in it being approved by FDA for use in treatment^110,111^. The combination therapy of docetaxel, a microtubule stabilizer and ramucirumab, a VEGF receptor 2 (VEGFR2) antagonist, proved to be effective in clinical trials conducted on patients with non-small-cell lung cancer (NSCLC) or gastric cancer and has been approved for treating NSCLC^112,113^.

The co-administration of paclitaxel and DNA-damaging agents reduces the efficiency of nonhomologous end joining and the phosphorylation of DNA-dependent protein kinase through microtubule disruption^114^. The effectiveness of a combination therapy of combretastatin A4 phosphate (CA4P), which targets tubulin and interferes with microtubule assembly, and carboplatin, which inhibits DNA synthesis by forming interstrand DNA crosslinks, was studied in patients with progressing solid tumors^115^. A three-drug combination therapy of paclitaxel, olaparib and durvalumab demonstrated high efficacy in patients with human epidermal growth factor receptor 2 (HER2)-negative breast cancer^116^. Olaparib induces DNA damage, whereas durvalumab suppresses PD-L1. A combination therapy of paclitaxel and carboplatin resulted in an increase in progression-free survival and overall survival in patients with NSCLC or breast cancer^117,118^.

### Drug delivery systems using liposomes and nanoparticles for cytoskeleton-targeting anticancer drugs

Liposome-based drug delivery systems are being developed to improve the efficacy and safety of treatment. Two such systems are currently in practice: targeted liposomes and stimuli-responsive liposomes (Table 2). Targeted liposomes can function as ligands through the attachment of antibodies or aptamers to their surface, thereby increasing the efficiency of drug delivery by selectively binding to receptors on cancer cells or specific tissues. A preclinical study confirmed that delivery of paclitaxel to the lung was enhanced when the drug was delivered using a complex wherein surfactant proteins were fused to the surface of liposomes^119^. This liposome–paclitaxel system has undergone clinical trials to treat various cancers^120,121^. Recently, the applications of stimuli-responsive liposomes have been studied. These liposomes release drugs in response to stimuli, such as pH, enzymes and temperature. A study designed transferrin receptor (TFR)-targeting liposomes coated with T7 peptide (HAIYPRH) or transferrin to increase their affinity for TFR, which is overexpressed in cancer cells. These liposomes were designed to be structurally disrupted at low pH or owing to phospholipase A2 activity inside cancer cells. Another study delivered vincristine using TFR-targeting liposomes, which released the drug through structural disruption^122^.

**Table 2:** Drug delivery system for cytoskeleton-targeting anticancer agents.

Drug	Mechanism and features	Clinical progress	Reference
Liposome–paclitaxel	Targeted liposome: selectively binds to receptors on cancer cells or specific tissues	Phase 3	^120,121^
TFR-targeting liposome–vincristine	TFR-targeting liposome (stimuli-responsive liposome): drug release by specific stimuli such as pH, temperature, ultrasound or enzymes Vincristine: binds to tubulin and inhibits microtubule formation	Preclinical study	^122^
PLGA–PEG–paclitaxel	PLGA–PEG copolymer: has great potential in drug delivery systems as a tumor-targeting carrier	Preclinical study	^123^
Dendrimer–paclitaxel	Dendrimer: type of nanostructured drug carrier	Preclinical study	^124^

In addition, the delivery of paclitaxel using polymer nanoparticles composed of poly lactic-co-glycolic acid-polyethylene glycol (PLGA–PEG) has been reported to inhibit cytoskeleton formation in preclinical studies^123^. Dendrimers, another type of drug delivery vehicle, are artificial polymers with a tree-like branching structure. A clinical study reported that ovarian cancer was suppressed when paclitaxel was treated with polypropyleneimine immunodendrimers^124^.

### ADC

Antibody–drug conjugates (ADCs) are combinations of cytotoxic drugs and antibodies that can bind to antigens specifically expressed on cancer cells (Table 3). The first ADC approved for targeting breast cancer was ado-trastuzumab emtansine, a combination of a microtubule inhibitor, emtansine and an anti-HER2 antibody, trastuzumab^125^. Disitamab vedotin comprises monomethyl auristatin E, which inhibits tubulin polymerization, and hertuzumab, which blocks HER2 receptor dimerization. The two moieties are linked by a maleimidocaproyl-valine-citrulline linker, which can be cleaved by proteases. Disitamab vedotin has been approved for treating metastatic gastric cancer^126,127^.

**Table 3:** Antibody-conjugated cytoskeleton-targeting drugs.

Drug	Mechanism and features	Clinical progress	Reference
Ado-trastuzumab emtansine	Trastuzumab: antibody targeting HER2 receptor Emtansine: binds to tubulin and inhibits microtubule assembly	FDA approved	^125^
Disitamab vedotin	Monomethyl auristatin E: inhibits tubulin polymerization Hertuzumab: inhibits HER2 receptor dimerization	FDA approved	^127^

## Conclusion and perspectives

The cytoskeletons play a dual role in tumor cell metastasis by providing scaffolds and regulating dynamic processes essential for cell motility and survival under mechanical stress. Actin filaments drive the formation of protrusive structures, such as lamellipodia, filopodia and invadopodia, which are essential for cell motility and ECM degradation during invasion. Rho-ROCK-related signaling plays a vital role in regulating actomyosin contractility, enabling cells to generate forces against the microenvironment. Microtubules coordinate with actin networks to stabilize these protrusions and drive cell movement. Dynamic regulators such as CAMSAPs enhance microtubule stability and ECM degradation, promoting cancer cell invasiveness and survival. Microtentacles improve tumor cell survival in circulation by facilitating adhesion to other cells or vessel walls, helping cells resist shear stress in the bloodstream. Intermediate filaments, particularly vimentin, confer mechanical resilience, allowing cells to deform without rupture and withstand compressive forces during EMT, intravasation and extravasation.

Traditionally, MTAs such as paclitaxel and vincristine have demonstrated potent anticancer effects, but their clinical use is often limited by nonspecific cytotoxicity toward normal cells. Therefore, one of the biggest challenges in the development of cytoskeleton-targeting anticancer drugs is the toxicity toward and adverse effects on normal cells. Studies are actively evaluating the effectiveness of combination therapies, ADCs and new drug delivery systems. Combination therapy using antiangiogenic agents, DNA-damaging agents or immune checkpoint inhibitors with MTAs can minimize toxicity by lowering the drug dosage while improving therapeutic efficacy. Cytoskeletal drug delivery using liposomes and nanoparticles can also be an efficient and safe cancer treatment method that selectively suppresses only tumors.

Targeting key regulators of cytoskeletal dynamics through combination therapies may offer new opportunities for metastasis-specific interventions that have minimal impact on normal cells. Furthermore, developing targeted delivery platforms, such as ADCs or nanocarriers, will be crucial for improving the therapeutic specificity and safety of cytoskeleton-targeting therapies.
